# Development of Screening Tools to Predict Medication-Related Problems Across the Continuum of Emergency Department Care: A Prospective, Multicenter Study

**DOI:** 10.3389/fphar.2022.865769

**Published:** 2022-07-06

**Authors:** Simone E. Taylor, Elise A. Mitri, Andrew M. Harding, David McD Taylor, Adrian Weeks, Leonie Abbott, Pani Lambros, Dona Lawrence, Dana Strumpman, Reyhan Senturk-Raif, Stephen Louey, Hamish Crisp, Emily Tomlinson, Elizabeth Manias

**Affiliations:** ^1^ Pharmacy Department, Austin Health, Heidelberg, VIC, Australia; ^2^ Emergency Department, Austin Health, Heidelberg, VIC, Australia; ^3^ Department of Critical Care, Melbourne Medical School, University of Melbourne, Parkville, VIC, Australia; ^4^ Faculty of Medicine, Dentistry and Health Sciences, University of Melbourne, Parkville, VIC, Australia; ^5^ Pharmacy Department, Western Health, Footscray, VIC, Australia; ^6^ Pharmacy Department, Barwon Health, Geelong, VIC, Australia; ^7^ Pharmacy Department, Northern Health, Epping, VIC, Australia; ^8^ Pharmacy Department, Eastern Health, Box Hill Hospital, Box Hill, VIC, Australia; ^9^ Pharmacy Department, Manly Hospital, Manly, NSW, Australia; ^10^ Pharmacy Department, Northern Beaches Hospital, Frenchs Forest, NSW, Australia; ^11^ Pharmacy Department, Prince of Wales Hospital, Randwick, NSW, Australia; ^12^ Pharmacy Department, Monash Health, Dandenong Hospital, Dandenong, VIC, Australia; ^13^ Pharmacy Department, Monash Health, Casey Hospital, Berwick, VIC, Australia; ^14^ Pharmacy Department, Launceston General Hospital, Launceston, TAS, Australia; ^15^ School of Nursing and Midwifery, Centre for Quality and Patient Safety Research, Institute for Health Transformation, Faculty of Health, Deakin University, Burwood, VIC, Australia

**Keywords:** emergency department, medication management, risk factors, patient transfer, workforce

## Abstract

**Background:** Medication-related problems (MRPs) occur across the continuum of emergency department (ED) care: they may contribute to ED presentation, occur in the ED/short-stay unit (SSU), at hospital admission, or shortly after discharge to the community. This project aimed to determine predictors for MRPs across the continuum of ED care and incorporate these into screening tools (one for use at ED presentation and one at ED/SSU discharge), to identify patients at greatest risk, who could be targeted by ED pharmacists.

**Methods:** A prospective, observational, multicenter study was undertaken in nine EDs, between July 2016 and August 2017. Blocks of ten consecutive adult patients presenting at pre-specified times were identified. Within 1 week of ED discharge, a pharmacist interviewed patients and undertook a medical record review to determine a medication history, patient understanding of treatment, risk factors for MRPs and to manage the MRPs. Logistic regression was undertaken to determine predictor variables. Multivariable regression beta coefficients were used to develop a scoring system for the two screening tools.

**Results:** Of 1,238 patients meeting all inclusion criteria, 904 were recruited. Characteristics predicting MRPs related to ED presentation were: patient self-administers regular medications (OR = 7.95, 95%CI = 3.79–16.65), carer assists with medication administration (OR = 15.46, 95%CI = 6.52–36.67), or health-professional administers (OR = 5.01, 95%CI = 1.77–14.19); medication-related ED presentation (OR = 9.95, 95%CI = 4.92–20.10); age ≥80 years (OR = 3.63, 95%CI = 1.96–6.71), or age 65–79 years (OR = 2.01, 95%CI = 1.17–3.46); potential medication adherence issue (OR = 2.27, 95%CI = 1.38–3.73); medical specialist seen in past 6-months (OR = 2.02, 95%CI = 1.42–2.85); pharmaceutical benefit/pension/concession cardholder (OR = 1.89, 95%CI = 1.28–2.78); inpatient in previous 4-weeks (OR = 1.60, 95%CI = 1.02–2.52); being male (OR = 1.48, 95%CI = 1.05–2.10); and difficulties reading labels (OR = 0.63, 95%CI = 0.40–0.99). Characteristics predicting MRPs related to ED discharge were: potential medication adherence issue (OR = 6.80, 95%CI = 3.97–11.64); stay in ED > 8 h (OR = 3.23, 95%CI = 1.47–7.78); difficulties reading labels (OR = 2.33, 95%CI = 1.30–4.16); and medication regimen changed in ED (OR = 3.91, 95%CI = 2.43–6.30). For ED presentation, the model had a C-statistic of 0.84 (95% CI 0.81–0.86) (sensitivity = 80%, specificity = 70%). For ED discharge, the model had a C-statistic of 0.78 (95% CI 0.73–0.83) (sensitivity = 82%, specificity = 57%).

**Conclusion:** Predictors of MRPs are readily available at the bedside and may be used to screen for patients at greatest risk upon ED presentation and upon ED/SSU discharge to the community. These screening tools now require external validation and implementation studies to evaluate the impact of using such tools on patient care outcomes.

## Introduction

Transitions from the community into the emergency department (ED), to a hospital ward or back to the community, are transitions associated with medication-related problems (MRPs) (Claydon-Platt et al., 2012; Roughead et al., 2016; Marotti et al., 2011; Cornish et al., 2005; Galvin et al., 2013). MRPs may contribute to ED presentations or occur due to care provided in ED, for example, initiating new medications without fully understanding patients’ medical and medication history.

Approximately one half of MRPs associated with the ED setting go unrecognized or unaddressed by non-pharmacist ED clinicians (Hohl et al., 2005; Cavin and Sen, 2005). Increasingly, pharmacists are smoothing medication-related transitions of care (Bond and Raehl, 2007; deClifford et al., 2007; Patanwala et al., 2011; Patanwala et al., 2012; Cesarz et al., 2013; Proper et al., 2015; Tong et al., 2016), although many more patients present to ED than can be seen by this workforce. Screening tools could assist in identifying patients at greatest risk for MRPs, who pharmacists could focus upon. Such tools should identify patients at risk for MRPs across the continuum of ED care, not only those contributing to ED presentation. They should be quick for non-pharmacists to administer, use readily available information relevant to the broad range of patients who present to ED and have simple parameter definitions to optimize inter-rater reliability. Good specificity and sensitivity are important to detect patients at risk for MRPs but not have sizable numbers of patients receiving an intervention (e.g., being seen by an ED pharmacist) that they do not require.

**FIGURE 1:**
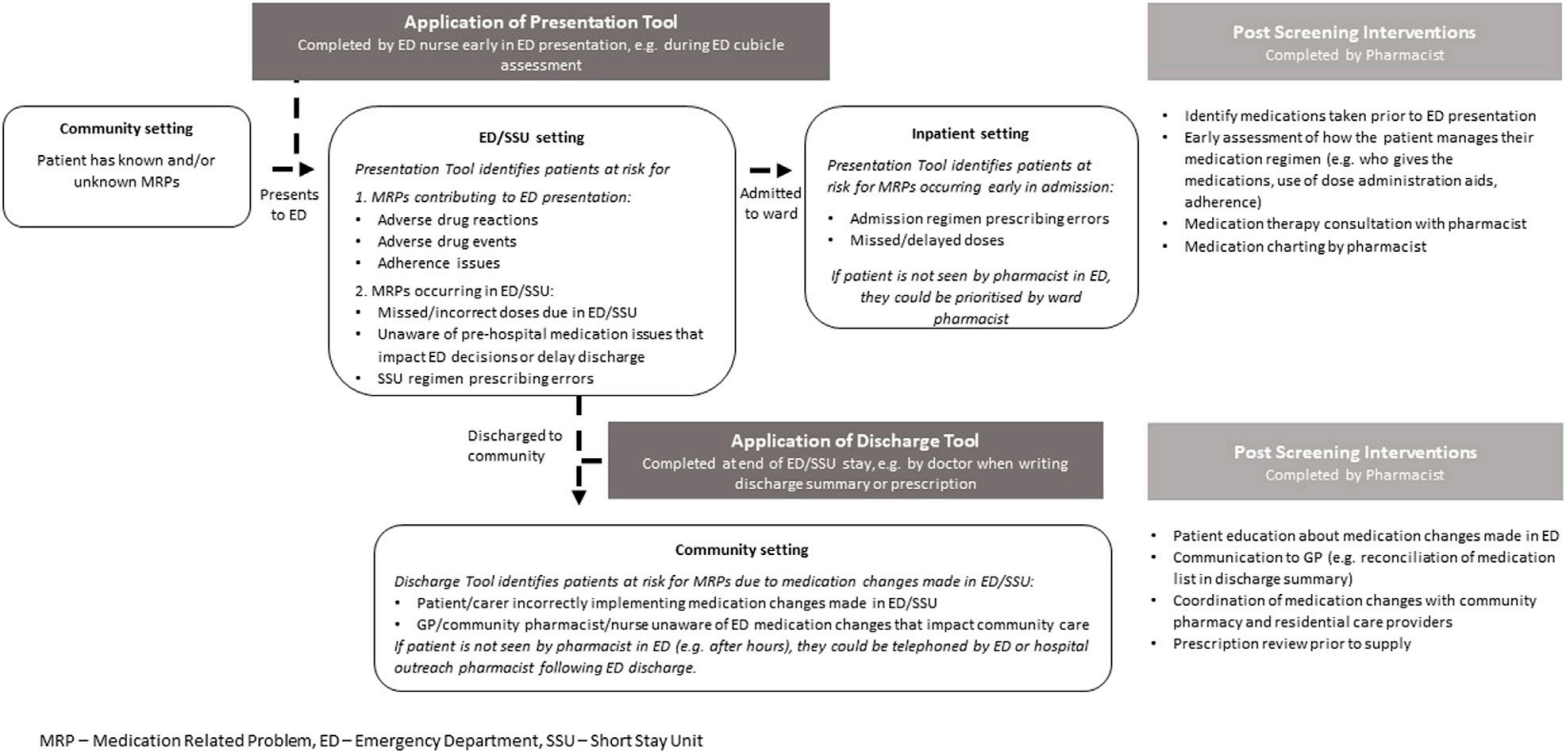
Medication-related problems occurring across the ED patient journey.

Several screening tools have been developed to identify patients at risk for MRPs. Some specifically assist in identifying patients with MRPs that contribute to the ED presentation (Hohl et al., 2005; Hohl et al., 2018). Others identify MRPs that occur when patients are admitted to hospital, but these often require pathology results and detailed past medical or medication history, which are time-consuming to accurately identify in ED (DeWinter et al., 2017; Parekh et al., 2020). Some tools are based upon expert opinion, rather than occurrence of actual MRPs (Kumar et al., 2011; Kaufmann et al., 2015). Our study aimed to develop two tools to identify patient, medication, and ED presentation related predictors for MRPs across the continuum of ED care that may require specialist input to identify, manage or prevent: at and during the ED presentation (Presentation Tool), and shortly after ED or short-stay unit (SSU) discharge (Discharge Tool) (Figure 1). The Presentation Tool could be used early in the ED presentation (e.g., by nurses during the ED cubicle assessment), to identify patients who could benefit from a specific focus on medications taken prior to presentation. Early identification of an accurate medication history and medication review could identify and manage medication-related contributors to the presentation, prevent patients from missing critical medications during their ED/SSU stay and advise on therapeutic decisions being made in ED. For those admitted to hospital, early review could ensure that the admission medication regimen is accurately prescribed. The Discharge Tool, to be used for patients returning to the community from ED/SSU, could detect patients at risk for MRPs related to medication regimen changes made in ED/SSU. Pharmacists could provide these patients with detailed medication education and ensure comprehensive clinical handover to community healthcare providers. As the two tools detect different types of MRPs, the relevant variables within each tool could differ.

## Materials and Methods

### Study Design and Setting

We undertook a prospective observational study in the EDs of nine Australian metropolitan and regional hospitals in the states of Victoria, New South Wales and Tasmania. Patient presentations to each ED in 2016 ranged from 25,000 to 92,000. Patient recruitment was undertaken between July 2016 and August 2017. The lead hospital ethics committee approved the study and each participating hospital provided governance approval before study commencement at each site.

### Patient Involvement

Patients were involved in piloting the data collection tool and informed the feasibility and acceptability of the study methodology. Patients and carers were interviewed after ED discharge to identify medication concerns and requirements for health professional follow-up.

### Selection of Participants

At each site, blocks of ten consecutive adult patients presenting to ED at pre-specified times across all days of the week were identified by pharmacist investigators. The times were determined randomly, prior to study commencement and covered the 24-h period. Patients were excluded if they did not wait to be seen by a clinician, were transferred from ED to another hospital, died in ED, a pharmacist was involved in their ED care or where it was deemed inappropriate to interview patients within 7 days of their presentation (e.g., severe mental health crisis). Patients interviewed face-to-face on a hospital ward did not provide consent as the medication review and data collection was undertaken as part of standard care. Patients discharged from ED or SSU provided verbal consent before undertaking the telephone interview.

### Development of the Data Collection Tool

Identification of the list of patient, medication-related and ED presentation variables that were potential predictors of MRPs was an iterative process. Four investigators (ST, AH, DT, EzM) drew on their extensive clinical practice experience in the ED and experience in undertaking medication safety research to derive an initial list. In developing the initial lists, investigators considered the resources produced by the Australian Commission on Quality and Safety in Health Care, specifically the classification of high-risk medicines and the Medication Risk Identification checklist of the Medication Management Plan (Australian Commission on Quality and Safety in Healthcare, 2013 and 2022). Two investigators (ST and EzM) undertook a narrative review of the literature for potential variables reported in previous studies (Claydon-Platt et al., 2012; Roughead et al., 2016; Marotti et al., 2011; Cornish et al., 2005; Galvin et al., 2013; Hohl et al., 2005; Cavin and Sen, 2005; Patanwala et al., 2011; Patanwala et al., 2012; Cesarz et al., 2013; deClifford et al., 2007; Kumar et al., 2011; Kaufmann et al., 2015; Saedeer et al., 2016; Fitzgerald et al., 2015). Related variables were grouped, then the four investigators worked together to come to a consensus as to the specific variables to include in the data collection tool. The literature search did not yield any additional variables over and above those initially identified by the investigators, however the literature search did assist with precisely defining variables and sparked discussion about the rationale for excluding variables that had been included in previous publications. Variables identified in the literature that were excluded, were excluded on the basis that they were imprecise (e.g., Kumar et al. included “other” under the list of comorbidities) or would be difficult to quickly measure at the bedside (e.g., variables with complex definitions, such as severity of organ dysfunction). Laboratory and diagnostic tests were avoided because not all ED patients require these tests routinely. Specific medications were not listed to avoid dating the screening tools as therapeutics evolve.

The data collection tool comprised three components: the first collected data from the hospital medical record (including information required by the pharmacist as part of the medication review and information required to measure some predictor variables), the second section included information that formed part of the pharmacists’ medication review to identify MRPs (documentation of a best possible medication history, identification of MRPs that required management) and the third section included a series of questions asked of the patient/carer to measure the predictor variables or confirm predictor variable information recorded in the medical record. Data were collected on 13 patient related variables including age, sex, presenting complaint, government benefit card status, social/living situation (living at home alone or with others) and cognitive and sensory issues. A total of 16 medication related variables were included, including the number and type of medications patients were taking prior to ED presentation, allergy status, who organizes the medications at home, medication adherence and what medications were prescribed in ED. Data were collected for 11 ED environment related variables, including triage category, the time of presentation, duration of ED stay and mode of presentation (e.g., *via* ambulance/emergency service or self-presenting). Further details are available in Supplementary Appendix S1. The data collection tool was piloted in 50 ED patient interviews, undertaken by an ED pharmacist at the lead site, before applying for ethical approval for the multisite study.

### Data Collection

Within 24–48 h after ED discharge, investigator pharmacists collected initial data from medical records. Following this, a patient and/or carer interview was undertaken by a pharmacist, face-to-face, for patients admitted to an inpatient ward, or *via* telephone, for patients discharged from ED/SSU to the community. If this interview could not be undertaken within 7 days of leaving ED, patients were deemed lost to follow-up. During the interview, data from the medical record review was verified, a best possible medication history was determined, patients’ understanding of ED medication regimen changes was assessed and a medication review was undertaken to identify, manage or prevent potential MRPs. Responses to a list of patient, medication-related and ED presentation variables that were potential predictors of MRPs was completed to ensure these were systematically recorded for each patient.

An MRP was defined as any medication error or adverse drug event that may require specialist input, such as an ED pharmacist, to identify, manage or prevent. Medication error was defined as “any preventable event that may cause or lead to inappropriate medication use or patient harm while the medication is in the control of the health care professional, patient or consumer” (National Coordinating Council for Medication Error Reporting and Prevention, 2022). Medication errors could occur at any stage of the medication management pathway, including the decision to prescribe, prescribing, dispensing, administration, monitoring or clinical handover to other health professionals. An adverse drug event was defined as an injury that occurred due to a medication; such adverse events could be preventable (e.g., due to a medication error) or non-preventable (e.g., idiosyncratic allergy). MRP types were classified according to the presence of a prescribing or administration error occurring prior to or in ED, an adverse drug event(s) or adverse drug reaction and/or presence of significant knowledge deficits and/or non-adherence to their prescribed medication regimen that may require specialist input to identify, manage or prevent (the specific types of MRPs are defined further in Supplementary Appendix S2). Two senior ED pharmacists independently reviewed all MRPs identified by investigator pharmacists during the patient interviews and medication reviews. MRPs were classified according to whether they could have been identified, managed or prevented by screening at ED presentation or ED discharge. MRP severity was classified according to a consequence-probability matrix (Society of Hospital Pharmacists of Australia, 2013). Discrepancies of opinion were resolved by consensus.

Examples of MRPs included in the ED presentation model were those that caused the presentation to ED, those that involved failure to prescribe and/or administer a time critical medication in ED (often a pre-admission medication that was not related to the reason for presentation but that had the potential to or did delay ED discharge if not given in a timely way) and prescribing errors on the hospital admission medication chart related to pre-admission medications. Examples of MRPs included in the ED discharge model included where a medication was initiated in ED that the patient was expected to take after leaving ED, but the patient failed to implement this change as intended. The implementation failure could be due to the patient not understanding the change that was intended, failing to have the medication dispensed or failure to handover medication information from ED to the general practitioner to assist with a smooth continuum of care.

### Primary Outcomes

The first primary outcome was the set of predictor variables that were significantly associated with MRPs that could be identified, managed, or prevented by evaluation of medication management at the time of ED presentation. This set informed the development of the Presentation Tool.

The second primary outcome was the set of predictor variables that were significantly associated with MRPs that could be identified, managed, or prevented at the time of ED/SSU discharge to the community. This set informed the development of the Discharge Tool.

### Data Analysis

We estimated that each site could recruit at least 100 patients. With a target sample size of 900, we would be 95% certain that the incidence of MRPs would lie ±1.8% of an incidence of 7.5% obtained in our pilot study. The precise number of patients recruited varied according to each site’s capability. The aim was to recruit more than 5 to 15 patients per explanatory variable (Tabachnick and Fidell, 1989); as the number of cases increased there was increased likelihood that the results obtained would be stabilized following regression analysis.

Statistical analysis was undertaken at the patient level. Univariate associations were examined between the presence of one or more MRPs and the patient, medication, and ED presentation-related predictor variables. Thirty variables were taken through to the multivariable regression analysis. Variables were excluded if there were difficulties collecting variables (due to >5% of missing data, or feedback from pharmacists that data was difficult to precisely collect during the interview) or if the prevalence was very low or if other variables captured similar information. Further details are provided in Supplementary Appendix S1. For the small amount of missing data, the more prevalent response was entered.

Multivariable logistic regression was undertaken using the backward Wald method recommended by Sun (Sun et al., 1996). Receiver operator characteristic (ROC) curves were constructed to determine the specificity and sensitivity of the models to predict MRPs. To identify weighted scores for screening tool predictor variables, beta-coefficients from the multivariable regression were multiplied by ten and rounded to the nearest whole number in a method used by Moore (Moore et al., 2012). Internal validation of the models was undertaken using bootstrapping of 1,000 resamples to assess reliability of the coefficients of regression (Danial et al., 2019). Standard errors were used to calculate the 95% bootstrap confidence intervals of the odds ratios. Data were analyzed using IBM SPSS (version 25).

## Results

### Characteristics of Study Subjects

Overall, 1730 patients were screened; 1,238 patients met all inclusion criteria, 277 were lost to follow-up and 57 patients declined consent. Demographic parameters for the 904 adult patients included 457 (50.6%) male, 134 (14.8%) aged 80 years and older, 292 (32.3%) brought to ED by an emergency service, and 409 (45.2%) taking four or more regular medications. Almost one third of patients (288, 31.9%) were hospitalized, whilst 616 (68.1%) were discharged from ED or SSU to the community.

One or more MRPs were identified during the pharmacist medication review in 381/904 (42.1%) patients. One or more MRPs of high, moderate, or low significance occurred in 60 (6.6%), 179 (19.8%) and 220 (24.3%) patients, respectively. High risk MRPs mostly involved high risk medications, particularly anticoagulants, strong opioids, and insulin. Further details have been published elsewhere (Taylor et al., 2020).

### Predictor Variables for Occurrence of MRPs Related to ED Presentation

One or more MRPs that could have been identified, managed, or prevented by screening early in the ED presentation were identified in 284/904 (31.4%) patients. The ED presentation was medication-related for 68 (7.5%) patients. The types of MRPs included in the ED presentation model are outlined in Table 1. One hundred and seventy-one (18.9%) patients had one or more MRPs classified as prescribing errors, whilst 155 (17.1%) had one or more MRPs classified as adherence or knowledge issues. Univariate associations between predictor variables and MRPs are detailed in Supplementary Appendix S3, Table 1.

**TABLE 1:** Types of medication-related problems included in the ED presentation and ED discharge models.

Type of MRP	Number of Patients with ≥1 of these MRPs overall^a^ (%) (n = 904)	Number of Patients with ≥1 of these MRP types included in ED presentation model^a^ (%) (n = 904)	Number of Patients with ≥1 of these MRP types included in ED discharge model^a^ (%) (n = 616)
Prescribing error	171 (18.9)	163 (18.0)	9 (1.4)
Adherence/knowledge issue	155 (17.1)	103 (11.4)	59 (9.6)
Adverse drug reaction	40 (4.4)	37 (4.1)	3 (0.4)
Drug-drug interaction	14 (1.5)	13 (1.4)	2 (0.3)
Medication administration error in ED	10 (1.1)	10 (1.1)	0 (0)
Clinical handover deficiency^b^	46 (5.1)	0	46 (7.5)
Other	12 (1.3)	8 (0.9)	4 (0.6)
Total number of patients with ≥1 MRP of any type	381 (42.1)	284 (31.4)	112 (18.2)

a Some patients had more than one type of problem or had problems included in the ED, presentation and ED, discharge models.

b Failure to inform general practitioner of significant prescription in ED, that patient was to take after discharge (for example, insulin, asthma inhalers, oxycodone, anticoagulant, antibiotic).

**FIGURE 2:**
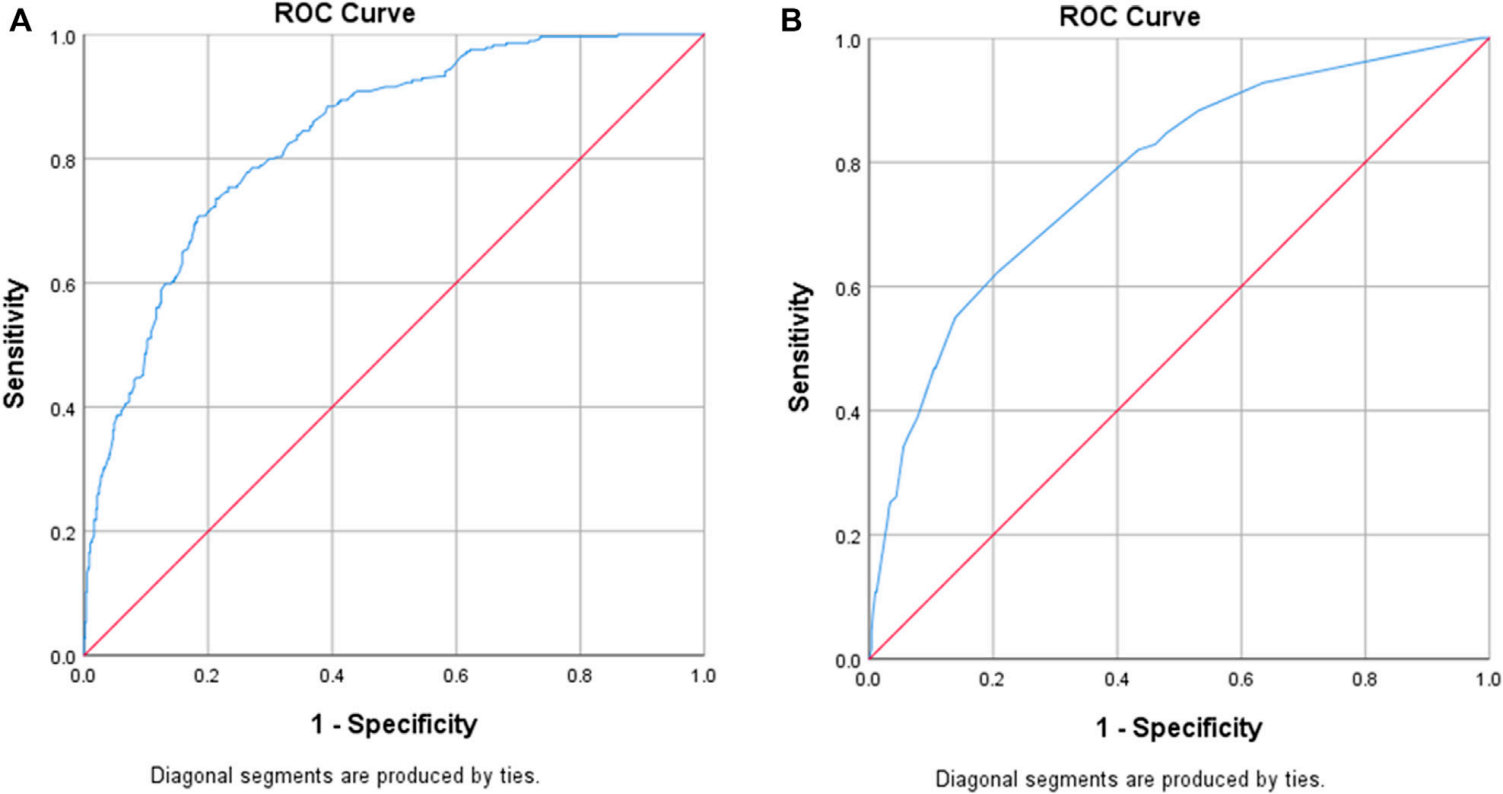
Receiver Operating Characteristic (ROC) curves for each model. **(A)** ED Presentation screening tool: AUC of ROC curve = 0.84 (95% CI = 0.81–0.86), [sensitivity 80%, specificity 70%]. **(B)** ED Discharge screening tool: AUC of ROC curve = 0.78 (95% CI =0.73–0.83), [sensitivity 82%, specificity 57%]

Significant predictors of MRPs in the multivariable logistic regression are summarized in Table 2. Eight predictor variables were significantly associated with increased risk of MRPs that could be addressed by screening at ED presentation: age, gender, pharmaceutical benefit (pension or concession) cardholder, who administers the medications at home, medication adherence, medication-related ED presentation, medical specialist seen recently and recent hospital admission. If patients had difficulty reading medication labels, this was protective for MRPs (OR = 0.63, 95%CI = 0.40–0.99). The ED presentation model provided an area under the curve (AUC) for the ROC curve of 0.84 (95% CI = 0.81–0.86). At a sensitivity of 80%, the model had a specificity of 70%, whilst at a sensitivity of 90%, specificity was 57% (Figure 2A).

**TABLE 2:** ED Presentation Screening Tool: summary of multivariable regression analysis of predictor variables for medication-related problems that could be identified/managed/prevented by screening early in the ED presentation (n = 904).

MRP Predictor variables	Odds ratio	95% confidence interval	Regression coefficient	Score assigned^1^
Medication related ED presentation	9.95	4.92–20.10	2.297	23
At home, medication administered by				
Self-administers	7.95	3.79–16.65	2.073	21
Carer assists	15.46	6.52–36.67	2.738	27
Health professional administers	5.01	1.77–14.19	1.611	16
No medications prior to ED	1.0	—	0	0
Patient age				
80 + years	3.63	1.96–6.71	1.289	13
65–79 years	2.01	1.17–3.46	0.699	7
40–64 years	1.60	0.97–2.65	0.472	5
18–39 years	1.0	-	0	0
Medication adherence Patient reports to sometimes or usually miss taking their medication doses	2.27	1.38–3.73	0.819	8
Seen a medical specialist in the past 6 months	2.02	1.42–2.85	0.701	7
Pharmaceutical benefit (pension/concession) card holder^2^	1.89	1.28–2.78	0.636	6
Recent admission: Inpatient in previous 4 weeks	1.60	1.02–2.52	0.472	5
Sex, male	1.48	1.05–2.10	0.394	4
Patient/carer who administers the medications has difficulties reading medication labels^3^	0.63	0.40–0.99	negative	0

1 Regression coefficient multiplied by 10 and rounded to the nearest whole number.

2 Pharmaceutical benefit card holders are those receiving income means tested Australian government benefits and entitles patients to more extensive medication cost subsidies than general patients.

3 The person who administers the medications has difficulties reading labels due to language barrier, intellectual difficulties, or visual acuity.

### Predictor Variables for Occurrence of MRPs Related to ED Discharge

One or more MRPs that could have been identified, managed, or prevented by screening at the time of ED/SSU discharge to the community were identified in 112/616 (18.2%) patients. The types of MRPs included in the ED discharge model are summarized in Table 1. Fifty-nine (9.6%) patients had one or more MRPs classified as adherence or knowledge issues, whilst 46 (7.5%) patients were noted to have inadequate clinical handover to the general practitioner. This included medications being prescribed in ED that the general practitioner was going to need to monitor or re-prescribe, where the general practitioner was not provided with the details as to what was prescribed in ED (for example, insulin, oxycodone, new anticoagulation or antiarrhythmics). Univariate associations between predictor variables and MRPs are detailed in Supplementary Appendix S3, Table 2.

Four variables were significant predictors of increased risk of MRPs that could be addressed by screening at ED discharge: patient adherence, difficulty reading medication labels, ED length of stay greater than 8 h and ED/SSU changes to the medication regimen (Table 3). The model for MRPs related to ED discharge provided an AUC for the ROC curve of 0.78 (95% CI = 0.73–0.83). At a sensitivity of 82%, specificity was 57% (Figure 2B).

**TABLE 3:** ED Discharge Screening Tool: summary of multivariable regression analysis of predictor variables for medication-related problems that could be identified/managed/prevented by screening at the time of ED discharge (n = 616).

MRP Predictor variables	Odds ratio	95% confidence interval	Regression coefficient	Score assigned^1^
Medication adherence Patient reports to sometimes or usually miss taking their medication doses	6.80	3.97–11.64	1.917	19
Medication regimen change in ED or short stay unit New medication started, pre-ED medication stopped or dose changed	3.91	2.43–6.30	1.363	14
ED length of stay				
>8 h	3.23	1.47–7.78	1.171	12
4–8 h	1.37	0.80–2.35	0.314	3
Patient/carer who administers the medications has difficulties reading medication labels^2^	2.33	1.30–4.16	0.845	8

1 Regression coefficient multiplied by 10 and rounded to the nearest whole number.

2 The person who administers the medications has difficulties reading labels due to language barrier, intellectual difficulties or visual acuity.

### Internal Validation

After conducting logistic regression with 1,000 sample bootstraps, results showed that the bootstrapping procedure did not change significant variables observed. Standard errors obtained for explanatory variables were similar to those obtained following bootstrapping, which indicated internal model validation.

### Weighted Scoring for Screening Tools

The beta coefficients and weighted scoring assigned for each predictor variable are reported in Tables 2 and 3. Tables 4 and 5 describe how these tools could be operationalized for use and scoring at the bedside. Potential scoring cut points and their corresponding sensitivity, specificity, positive predictive value (PPV) and negative predictive value (NPV) are also described.

**TABLE 4:** ED Presentation medication-related problem screening tool.

Question	Potential response	Score
Patient age	o 18–39 years	0
	o 40–64 years	5
	o 65–79 years	7
	o ≥ 80 years	13
Patient sex	o Female	0
	o Male	4
Pension or concession card holder? (Do they pay the pension/concession amount for their community prescriptions?)	o No	0
	o Yes	6
Who administers the medications at home?	o No regular medications taken at home	0
	o Patient themselves	21
	o Family, friend or carer helps	27
	o Health professional e.g., nurse	16
Is the ED presentation potentially medication-related (e.g., allergy, side effect, overdose, poor adherence)?	o No	0
	o Yes	23
Is there a potential medication adherence problem? “People often have difficulty taking their pills for one reason or another. How often do you miss taking a dose of your medicines?”	o No (Never/rarely/very occasionally/doesn’t take medicines)	0
	o Yes (Sometimes/Usually)	8
Has the patient visited a medical specialist as an outpatient in the last 6 months? (e.g., surgeon, cardiologist, psychiatrist, doctor other than their local doctor)?	o No	0
	o Yes	7
Recent admission: Was the patient in hospital within the past 4 weeks?	o No	0
	o Yes	5

**Score cut-off**	**Sensitivity (95%CI)**	**Specificity (95%CI)**	**Positive predictive value (95%CI)**	**Negative predictive value (95%CI)**
30 or less/Greater than 30	0.90 (0.86–0.93)	0.55 (0.51–0.59)	0.48 (0.44–0.52)	0.92 (0.89–0.95)
40 or less/Greater than 40	0.63 (0.57–0.69)	0.83 (0.80–0.86)	0.63 (0.57–0.68)	0.83 (0.80–0.86)

95%CI, 95% confidence interval.

**TABLE 5:** ED Discharge medication-related problem screening tool.

Question	Response	Score
ED length of stay: Duration of stay in ED? (excluding short stay unit)	o Up to 4 h	0
	o Between 4–8 h	3
	o More than 8 h	12
Medication regimen change: In ED/short stay unit, was a new medication started, a pre-ED medication stopped or dose changed?	o No	0
	o Yes	14
Reading difficulties: Does the patient (or the person who helps with the medication routine) have difficulty reading medication labels?	o No	0
	o Yes	8
Is there a potential medication adherence problem? “People often have difficulty taking their pills for one reason or another. How often do you miss taking a dose of your medicines?”	o No (Never/rarely/once in a while/doesn’t take medicines)	0
	o Yes (Sometimes/Usually)	19

**Score cut-off**	**Sensitivity (95%CI)**	**Specificity (95%CI)**	**Positive predictive value (95%CI)**	**Negative predictive value (95%CI)**
12 or less/Greater than 12	0.72 (0.63–0.80)	0.57 (0.53–0.62)	0.27 (0.22–0.33)	0.90 (0.86–0.93)
20 or less/Greater than 20	0.36 (0.27–0.45)	0.90 (0.87–0.93)	0.45 (0.35–0.56)	0.86 (0.83–0.89)

95%CI, 95% confidence interval.

For the Presentation Tool, potential scores range from a minimum of 0 and to a maximum of 93. Using the scoring approach outlined in Table 4, the median (interquartile range) score in the derivation dataset was 34 (18–44). Using a score cut-off score of above 30, the sensitivity, specificity, PPV and NPV, with associated 95% confidence intervals were 0.90 (0.86–0.93), 0.55 (0.51–0.59), 0.48 (0.44–0.52) and 0.92 (0.89–0.95), respectively.

For the Discharge Tool, potential scores range between a minimum of 0 and maximum of 53. Using the scoring approach outlined in Table 5, the median (interquartile range) score was 12 (0–14). Using a score cut-off score of above 12, the sensitivity, specificity, PPV and NPV, with associated 95% confidence intervals were 0.72 (0.63–0.80), 0.57 (0.53–0.62), 0.27 (0.22–0.33) and 0.90 (0.86–0.93), respectively.

## Discussion

### Statement of Principal Findings

Key predictor variables for MRPs that could be identified, managed, or prevented by screening at the time of ED presentation and as patients were discharged from ED/SSU to the community have been identified. These predictor variables are readily collected at the bedside and have been incorporated into two screening tools to capture patients at risk for MRPs across the ED continuum of care. A weighted scoring system has been developed and using some preliminary score cut-points, the scoring tools’ performance characteristics are reported. Overall, the models have similar predictive characteristics to other published models (Hohl et al., 2018; DeWinter et al., 2017; Kumar et al., 2011; Kaufmann et al., 2015; Geeson et al., 2019), but either screen for a broader range of MRPs or are more practical for ED use.

### Presentation Tool MRP Predictors

Increasing patient age was associated with increasing risk of MRPs, which is consistent with previous studies (DeWinter et al., 2017; Geeson et al., 2019; Parekh et al., 2020). Being older and very much older were associated with increased risk of MRPs, independent of the number of medications taken before presentation.

Several predictors related to patients’ ability to manage their medications at home and to communicate their medication history in ED. Patients with carers assisting with medication administration were at particularly high risk for MRPs related to ED presentation. High-risk medications were significant predictors in the univariate analysis but did not remain significant in the multivariable analysis. These high-risk medications may not predict a patients’ risk of MRPs if they are capable of accurately articulating to a health professional how they take these medications at home. If the carer who assists with medication administration is not available in ED, it may be difficult for ED clinicians to accurately elicit this history, thus putting this patient group at higher risk of MRPs (WHO, 2014).

Being a government pharmaceutical benefit cardholder may be a marker of socioeconomic status. One published screening tool excluded socioeconomic status because it was difficult to measure reliably (Geeson et al., 2019). Some markers of socioeconomic status are confronting for health professionals and may not be appropriate to ask in ED. Patients are routinely asked about their benefit status when a community prescription is dispensed, therefore this may be a feasible method to identify this potential predictor of MRPs.

The person who administers the medications at home having difficulties reading medication labels being a protective factor for having an MRP related to presentation was unexpected. The upper level of the 95% confidence interval for the odds ratio was 0.99, therefore this variable is at the margin of our definition of a variable that would be retained within the multivariable model. It is possible that this variable may fall outside of the criteria for inclusion in a future validation sample. If this variable is retained within the model, it is possible that patients and carers who are aware of their difficulties reading labels may take more care and use other resources to minimize the risk of medications errors.

### Discharge Tool MRP Predictors

Key predictors of MRPs relevant to patients being discharged from ED/SSU to the community were whether there was a medication regimen change made in ED/SSU that the patient needed to implement, whether there was evidence of poor adherence and whether they had difficulties reading medication labels (due to English language, intellectual or visual acuity problems of the person administering the medications). In addition, longer duration of ED stay, may indicate a more complex presentation or presentation at a time when the ED capacity was stretched such that staff were unable to provide adequate discharge education or clinical handover.

### Interpretations Within the Context of the Wider Literature

Kumar (Kumar et al., 2011) developed an ED pharmacist referral tool in their emergency SSU using patient characteristics based upon expert-panel opinion. Their tool identified patients at risk for MRPs across the continuum of ED care. Patients with a medication-related presentation; newly prescribed warfarin; over 70 years, taking five or more medications, and with three or more comorbidities, were identified to be at high risk for medication misadventure. This tool had good levels of specificity and sensitivity of 78 and 83%, respectively. However, whilst the list of comorbidities was pragmatic, it is infinite, and the comorbidities were poorly defined. Warfarin use is declining as newer options become available; specifying particular medications within a tool has the potential to date the tool as therapy evolves. During routine ED care, it is not possible to systematically identify all potential comorbidities and whether they are active issues, therefore, co-morbidities were not included in our models.

Two decision rules were developed in three Canadian EDs, to identify patients presenting to ED with moderate/severe adverse drug events (ADEs) (Hohl et al., 2018). The following factors were associated with presentation with ADEs: rule 1 comprised having a pre-existing medical condition or having taken antibiotics within 1 week of presentation; rule 2 comprised age over 80 years or having a medication change within 28 days. These rules would be practical to administer in the ED, but only detected those patients at risk for presenting to ED with an MRP. The rules had a sensitivity of 91.3% and specificity of 37.9%. At a sensitivity of 80%–82%, our models have greater specificity (57%–70%), although ideally our models would also have greater specificity. With low levels of specificity, some patients may be unnecessarily seen by a pharmacist, which has workforce implications.

A prospective study undertaken on adult medical wards of two United Kingdom hospitals (Geeson et al., 2019) developed a 12-item prognostic model to prevent MRPs of at least moderate severity, with a sensitivity of 90% and a specificity of 30%. The model included the number of regular medications prescribed on the first full day of admission, which is not feasible for ED patient screening. It included pathology results to estimate renal function and white cell counts, which are not universally measured in ED patients.

A study by DeWinter et al. (2017) developed a decision rule to identify which admitted patients needed medication reconciliation. This rule only identified MRPs in admitted patients, rather than considering MRPs across the continuum of ED care. It did not include patients discharged from ED to the community, who comprise the greatest proportion of patients who present to an ED. Administering the rule required detailed knowledge of medication groups taken by patients, which would be time-consuming to complete during an ED cubicle assessment. Likewise, a rigorously designed United Kingdom study identified hospitalized patients at risk for MRPs (Kaufmann et al., 2015). They used a mixed-methods approach comprising a literature search and expert-panel using the nominal group technique. Eighty-five risk factors for MRPs were narrowed to 27 judged to be ‘important’ or ‘rather important’. Accurately gathering this number of variables in ED would be problematic, even if this tool could be automated.

### Implications for Policy, Practice, and Research

The screening tools developed in relation to this study may assist ED pharmacists to ensure they see higher-risk patients, may help ward staff to prioritize patients for early ward review, and highlight to ED nurses and doctors, which patients need greater medication-related support at or shortly following ED discharge.

Predictors in both models are amenable to being incorporated into electronic patient management systems with some auto-populated information. Some parameters will need ED clinicians to check-off, such as who administers the medications at home and how often medication doses are missed. Once completed, pharmacist follow-up could be electronically triggered (DeWinter et al., 2017; Geeson et al., 2019). At risk patients identified outside of clinical pharmacy hours could be followed up by telephone after ED discharge or be prioritized to be seen by ward pharmacists. The tool score cut-off points could be varied depending upon the availability of the pharmacist workforce to follow-up patients identified to be at risk.

Tool validation is required in indigenous populations, private hospital ED patients and hospitals with poorly developed clinical pharmacy services. Although speculative, indigenous patients may require additional variables to be included, such as whether they live remotely or in a metropolitan area. In addition, the age categories may need to be reduced to younger years of age as is required for several health interventions in this population, such as vaccination eligibility (Australian Technical Advisory Group on Immunisation (ATAGI), 2018) and interventions for cardiovascular disease (Reath and O’Mara, 2018).

The PROGRESS framework (Steyerberg et al., 2013) for prognosis research outlines the stepwise process for the development and evaluation of prognostic or predictive tools. This stepwise process involves model development, followed by external validation of the model using a new dataset, then impact evaluation to assess the impact of tool implementation on health outcomes. Our study describes the initial step in this process. External, prospective validation and impact evaluation are required to determine the performance of the tools in practice. Also, assessment of inter-rater reliability is required.

### Strengths and Limitations

The tools were developed using multicenter prospective data and outcomes relevant for patients and clinicians. To minimize selection bias but also maintain the depth of medication review for each patient (to optimize data accuracy and completeness), blocks of ten consecutive adult ED patients who presented to a range of EDs, at different times of the day across 7 days of the week were included. MRPs associated with ED care were identified for patients discharged from ED as well as those who were hospitalized. Using an objective approach to patient recruitment, rather than only those seen by ED pharmacists minimizes selection bias and enables identification of patients at risk for MRPs, and those not at risk. Predictors are readily determined at the bedside. By not including specific medications in the tools, a detailed medication history is not required at the point of screening, and the tools are less prone to becoming dated as medication prescribing practices evolve.

Some patients were lost to follow-up, particularly those discharged directly from ED. The tools may not identify all patients likely to benefit from clinical pharmacist review, e.g., patients with sepsis where an ED pharmacist could facilitate timely provision of the first antibiotic dose (Roman et al., 2018). MRPs due to dispensing and administration errors may have been under-estimated if not documented during the ED presentation. MRPs related to patient/carer knowledge deficits and non-adherence are likely under-estimated, as these MRPs were identified during the pharmacist interview/review in a process that mirrored routine care, rather than using specific tools validated to identify patient knowledge and adherence issues. The definition required that a knowledge deficit be one where the patient may be harmed by the knowledge deficit, therefore only the most serious knowledge deficits were included.

The data collection process, involving experienced pharmacists undertaking comprehensive medication reviews and reconciling data with several sources of information maximized the completeness of the data collection process. For the majority of variables taken through to the multivariable regression analysis there were no missing data. Of over 27,000 pieces of data taken through to the multivariable regression analysis there was a total of 103 pieces of missing data. The variable with the greatest prevalence of missing data was whether a medical specialist had been seen in the previous 6 months (there were 35 (3.9%) patients missing this data element). For patients missing this data variable, patients had often seen a specialist but found it difficult to recall whether it was within 6 months or within the past 6–12 months. So as not to over-estimate the potential risk, patients with missing data were coded as not having seen a specialist within the previous 6 months (this was also the most prevalent response). All patients had a comprehensive assessment of the MRP outcome variables and no patient had missing outcome data.

Ideally the tools would have greater specificity, but this highlights the broad range of ED MRPs and their multifactorial etiology. Preliminary scoring cut-points and associated screening tool performance have been proposed using this derivation dataset. These performance outcomes need to be further evaluated using a separate validation dataset.

## Conclusion

In conclusion, predictors of MRPs that are readily available within the ED have been identified and built into tools to screen for patients at greatest risk for MRPs across the ED continuum of care. Future studies are required to prospectively validate these tools and evaluate their impact in practice.

## Data Availability

The data underlying this article may be shared on reasonable request to the corresponding author and approval of the approving Human Research and Ethics Committee.
