# Supplementary material for: Development of Screening Tools to Predict Medication-Related Problems Across the Continuum of Emergency Department Care: A Prospective, Multicenter Study
Source: Front Pharmacol. 2022 Jul 6;13:865769. doi: 10.3389/fphar.2022.865769 (PMC9299090; doi:10.3389/fphar.2022.865769)
Supplement: Supplementary file 1 [file DataSheet2.PDF]

Appendix 2: Definitions of types of medication related problems

1. Prescribing errors:

- May occur on the admission medication chart.
    - Detection of such errors by a pharmacist led to a change to the patient's medication regimen in collaboration with the inpatient medical team.
- May occur when patients are discharged directly from the ED/SSU to the community.
    - Such errors were defined as those identified or corrected by the pharmacist during the telephone follow-up, in collaboration with the patient, community pharmacist and/or general practitioner (GP).
- Prescribing errors comprised wrong or omitted medication, dose or frequency; unnecessary treatment (ceased medication charted); wrong form or route of administration, and wrong patient.

2. Adverse drug reactions:

- Defined as a response to a medication, which was harmful and unintended and occurred at normal doses, i.e. the right medication was used for the right indication in the right dose and route but the patient still suffered harm.

3. Administration errors:

- The correct regimen was prescribed but an incorrect regimen was administered. These included an omitted dose or an extra dose, wrong time, administering to the wrong patient, wrong infusion rate, wrong route (different from that prescribed), wrong technique, wrong dosage-form or preparation.

4. Drug-drug interactions:

- These were those interactions deemed clinically significant or having the probability of harm according to the evidence-based guidelines specified in the Australian Medicines Handbook, Lexicomp Online and/or Stockley's Drug Interactions.

5. Significant knowledge deficit:

- Patients discharged from ED/SSU were asked to state the name, dose regimen or how to take/administer the medication and the indication for any newly prescribed medication. Where the patient taking the medication regimen, or the carer responsible for giving the regimen after ED/SSU discharge, was unaware of the significant aspects of the medication taking process for a new medication prescribed in ED/SSU, to be taken following ED/SSU discharge back to the community, this was defined as an MRP. Significant aspects were those deficits where patients could experience harm due to their lack of knowledge, e.g. incorrect number of doses of glyceryl trinitrate prior to calling an ambulance.
- Knowledge was not assessed for patients admitted to an inpatient ward who were prescribed medications in ED, as these medications could change during the inpatient admission and the ED was not considered the optimal time to teach patients being admitted to a ward about new medications.

6. Demonstrated non-adherence:

- Where non-adherence to the medication regimen was identified during the medication review/interview, that could potentially have contributed to the need for the ED presentation or other acute medical follow-up after ED discharge, this was deemed an MRP.

7. Clinical handover issue:

- Where a significant change was made to a patient's medication regimen in ED that the GP should be aware of so that they can provide optimal ongoing care, the ED doctor should document this in the ED discharge summary.
- If an ED doctor failed to inform the GP that a short-term, minor symptomatic treatment was started, such as a simple analgesic or antacid, this was not considered significant. Those that were considered significant were failure to notify that an antibiotic or strong opioid was prescribed, or a medication was initiated or dose changed that the GP may need to further titrate, such as insulin or an antihypertensive.

Classification of medication related problems according to when they could be identified, managed or prevented

ED Presentation:

- MRPs that occurred prior to ED presentation (and related/contributed to the presentation), or occurred in ED that could have been identified, managed or prevented had a medication review been undertaken early in the ED presentation.
- Examples:
    - Adverse drug reaction or poor adherence prior to ED presentation that contributed to the ED presentation.
    - Failure to continue time critical regular medications during the ED presentation, such as insulin, anti-parkinsonian medications or antihypertensives.
    - Prescribing errors on the hospital admission medication chart for patients admitted from ED to an inpatient ward.

ED discharge:

- Those MRPs that could be managed or prevented by screening the patient at ED/SSU discharge.*
- Examples:
    - Changes were made to the medication regimen at ED/SSU discharge that the patient did not implement after leaving ED/SSU.
    - This lack of implementation may have been due to inadequate patient education or inadequate continuum of care planning, such as notifying a community pharmacy that the regimen in the dose administration aid that they filled had changed.

***** If MRPs were detected at the post discharge interview due to care provided since leaving ED, these were not included as ED discharge-related MRPs.
