# Supplementary material for: Development of Screening Tools to Predict Medication-Related Problems Across the Continuum of Emergency Department Care: A Prospective, Multicenter Study
Source: Front Pharmacol. 2022 Jul 6;13:865769. doi: 10.3389/fphar.2022.865769 (PMC9299090; doi:10.3389/fphar.2022.865769)
Supplement: Supplementary file 2 [file DataSheet4.PDF]

Appendix 3

Table 1 – Patient, medication-related and ED presentation variables associated with medication-related problems that could be managed by screening early in the ED presentation

Variable	Total sample (n=904)	Univariate OR (95% CI)	Univariate p	Multivariable OR (95% CI)	Multivariable p
Patient characteristics					
Age category					
80 years and older	134 (14.8)	8.12 (5.04 – 13.10)	<0.01	**3.63 (1.96 – 6.71)**	**< 0.01**
65 – 79 years	203 (22.5)	5.04 (3.27 – 7.77)	<0.01	**2.01 (1.17 – 3.46)**	**0.01**
40 – 64 years	279 (30.9)	2.36 (1.55 – 3.61)	<0.01	1.60 (0.97 – 2.65)	0.07
18 – 39 years	288 (31.9)	1			
Sex					
Male	457 (50.6)	1.40 (1.06 – 1.86)	0.02	**1.48 (1.05 – 2.10)**	**0.03**
Female	447 (49,4)	1			
Pharmaceutical benefit cardholder*					
Yes	407 (45.0)	3.28 (2.44 – 4.39)	<0.01	**1.89 (1.28 – 2.78)**	**<0.01**
No	497 (55.0)	1			
Private health insurance					
No	665 (73.6)	1.06 (0.77 – 1.46)	0.74	1.04 (0.69 – 1.58)	0.84
Yes	239 (26.4)	1			
Interpreter required in ED					
Yes	70 (7.7)	1.61 (0.98 – 2.64)	0.06	0.77 (0.39 – 1.52)	0.45
No	834 (92.3)	1			
Oral communication difficulties - medical or language reasons					
Unable to describe medication history in English	207 (22.9)	2.47 (1.79 – 3.40)	<0.01	1.32 (0.80 – 2.18)	0.28
Able to describe medication history in English	697 (77.1)	1			
Inpatient within previous 4 weeks					
Yes	135 (14.9)	2.51 (1.73 – 3.64)	<0.01	**1.60 (1.02 – 2.52)**	**0.04**
No	769 (85.1)	1			
Seen medical specialist in past 6 months					
Yes	383 (42.4)	2.73 (2.04 – 3.64)	<0.01	**2.02 (1.42 – 2.85)**	**<0.01**
No	521 (57.6)	1			
ED presentation within the past month					
Yes	173 (19.1)	1.85 (1.32 – 2.61)	<0.01	1.03 (0.61 - 1.74)	0.92
No	731 (80.9)	1			
Medication characteristics					
Medication-related problem contributed to ED presentation					
Yes	68 (7.5)	12.44 (6.55–23.64)	<0.01	**9.95 (4.92 – 20.10)**	**<0.01**
No	836 (92.5)	1			
Number of regular medications taken prior to presentation					
More than 8 medications	171 (18.9)	30.74 (14.80–63.82)	<0.01	8.54 (0.18 – 398.69)	0.27
4-8 medications	238 (26.3)	22.91 (11.23–46.74)	<0.01	7.25 (0.16 – 334.94)	0.31
1 – 3 medications	254 (28.1)	9.81 (4.77 – 20.16)	<0.01	5.48 (0.12 – 248.24)	0.38
No medications	241 (26.7)	1			
At home medications administered by…					
Self-administers	519 (57.4)	15.31 (7.69–30.51)	<0.01	**7.95 (3.79 – 16.65)**	**< 0.01**
Carer helps	103 (11.4)	38.48 (17.74–83.44)	<0.01	**15.46 (6.52 – 36.67)**	**< 0.01**
Health professional administers	44 (4.9)	17.62 (7.18–43.20)	<0.01	**5.01 (1.77 – 14.19)**	**<0.01**
No medications taken prior to presentation	238 (26.3)	1			
Self-reported medication allergies/ sensitivities/intolerances that should be avoided or re-prescribed with caution					

Yes	130 (14.4)	1.75 (1.19 – 2.55)	<0.01	1.54 (0.98 – 2.43)	0.06
No	774 (85.6)	1			
Person who organises the medications has difficulty…					
Reading medication labels	159 (17.6)	1.46 (1.02 – 2.08)	0.04	**0.63 (0.40 – 0.99)**	**0.04**
No problems reading labels	745 (82.4)	1			
Opening medication bottles	94 (10.4)	2.42 (1.57 – 3.72)	<0.01	1.35 (0.79 – 2.29)	0.28
No problems opening bottles	810 (89.6)	1			
Medication adherence					
Sometimes/usually misses doses	117 (12.9)	2.08 (1.40 – 3.08)	<0.01	**2.27 (1.38 – 3.73)**	**<0.01**
Never, rarely, very occasionally misses doses	787 (87.1)	1			
Taking high risk medication prior to presentation					
Yes	230 (25.4)	3.22 (2.36 – 4.41)	<0.01	1.10 (0.70 – 1.73)	0.68
No	674 (74.6)	1			
Taking an anticoagulant prior to presentation					
Yes	68 (7.5)	2.85 (1.73 – 4.69)	<0.01	0.81 (0.44 – 1.48)	0.49
No	836 (92.5)	1			
Taking insulin prior to presentation					
Yes	48 (5.3)	1.75 (0.97 – 3.16)	0.06	0.84 (0.40 – 1.78)	0.65
No	856 (94.7)	1			
Taking regular strong opioid prior to presentation					
Yes	70 (7.7)	2.35 (1.44 – 3.84)	<0.01	0.84 (0.42 – 1.69)	0.62
No	834 (92.3)	1			
Prescription/s dispensed at more than one pharmacy in previous 6 months					
Yes	218 (24.1)	0.99 (0.71 – 1.37)	0.94	1.09 (0.71 – 1.65)	0.70
No	686 (75.9)	1			
ED environment/presentation characteristics					
Time of presentation,					
Out of ‘office hours’	592 (65.5)	0.98 (0.73 – 1.31)	0.88	1.01 (0.70 – 1.47)	0.95
During ‘office hours’ (9am – 5pm)	312 (34.5)	1			
Triage category (Australasian Triage Scale)					
1 (seen immediately)	4 (0.4)	1.70 (0.17 – 16.80)	0.65	2.00 (0.13 – 30.04)	0.62
2 (seen within 10 minutes)	130 (14.4)	1.48 (0.15 – 14.35)	0.74	1.60 (0.11 – 23.40)	0.73
3 (seen within 30minutes)	418 (46.2)	1.10 (0.11 – 10.74)	0.93	1.66 (0.11 – 24.54)	0.71
4 (seen within 60 minutes)	298 (33.0)	1.50 (0.15 – 15.46)	0.73	3.88 (0.25 – 61.46)	0.34
5 (seen within 120 minutes)	54 (6.0)	1			
Mode of presentation					
Ambulance/Emergency service	292 (32.3)	2.23 (1.66 – 2.99)	<0.01	0.95 (0.63 – 1.44)	0.81
Self	612 (67.7)	1			

Note: OR (95% confidence intervals) in bold are significant predictors of MRPs in the multivariable model.
* Pharmaceutical benefit card holders are those receiving income means tested Australian government benefits and entitles patients to more extensive medication cost subsidies than general patients.

Table 2: Patient, medication-related and ED presentation variables associated with medication-related problems that could be managed by screening at the time of ED discharge

Variable	Total sample (n=616)	Univariate OR (95% CI)	Univariate p	Multivariate OR (95% CI)	Multivariate p
Patient characteristics					
Age category					
80 years and older	54 (8.8)	0.69 (0.28 – 1.74)	0.44	0.93 (0.27 – 3.18)	0.91
65 – 79 years	127 (20.6)	1.10 (0.61– 1.98)	0.75	1.04 (0.45 – 2.38)	0.93
40 – 64 years	199 (32.3)	1.77 (1.09 – 2.86)	0.02	1.36 (0.75 – 2.49)	0.32
18 – 39 years	236 (38.3)	1			
Sex					
Male	312 (50.6)	1.62 (1.06 – 2.46)	0.03	1.42 (0.90 – 2.26)	0.14
Female	304 (49.4)	1			
Pharmaceutical benefit card holder*					
Yes	232 (37.7)	1.27 (0.84 – 1.93)	0.26	1.09 (0.61 – 1.94)	0.78
No	384 (62.3)	1			
Private health insurance					
No	444 (72.1)	1.06 (0.67 – 1.68)	0.82	0.93 (0.54 – 1.60)	0.80
Yes	172 (27.9)	1			
Interpreter required in ED					
Yes	35 (5.7)	1.63 (0.74 – 3.57)	0.23	1.75 (0.67 – 4.52)	0.25
No	581 (94.3)	1			
Oral communication difficulties for medical or language reasons					
Unable to describe medication history in English	106 (17.2)	1.24 (0.74 – 2.09)	0.42	0.90 (0.40 – 2.02)	0.80
Able to describe medication history in English	510 (82.8)	1			
Seen by a medical specialist in the previous 6 months					
Yes	237 (38.5)	1.01 (0.67 – 1.55)	0.95	0.94 (0.55 – 1.61)	0.83
No	379 (61.5)	1			
Medication characteristics					
Number of regularly scheduled medications taken prior to presentation					
More than 8 medications	82 (13.3)	0.77 (0.36 – 1.65)	0.49	0.46 (0.15 – 1.46)	0.19
4-8 medications	145 (23.5)	1.38 (0.79 – 2.41)	0.26	1.12 (0.54 – 2.34)	0.76
1 – 3 medications	187 (30.4)	1.55 (0.93 – 2.60)	0.10	1.18 (0.64 – 2.16)	0.60
No medications	202 (32.8)	1			
At home medications administered by…					
Self-administers	352 (57.1)	1.34 (0.84 – 2.13)	0.22	0 (0.00 – 0.00)	1.00
Carer helps	42 (6.8)	1.47 (0.64 – 3.37)	0.36	0 (0.00 – 0.00)	1.00
Health professional administers	24 (3.9)	0.23 (0.03 – 1.80)	0.16	0 (0.00 – 0.00)	1.00
No medications taken prior to presentation	198 (32.1)	1			
Dose administration aid used					
Yes, packed by self or carer	59 (9.6)	0.90 (0.44 – 1.83)	0.76	0.56 (0.25 – 1.28)	0.17
Yes, packed by health professional	40 (6.5)	0.63 (0.24 – 1.64)	0.34	0.44 (0.15 – 1.32)	0.14
No	517 (83.9)	1			
Person who organizes the medications has difficulty…					
Reading medication labels	87 (14.1)	2.08 (1.24 – 3.49)	<0.01	**2.33 (1.30 – 4.16)**	**<0.01**
No problems reading labels	529 (85.9)	1			
Opening medication bottles	50 (8.1)	1.31 (0.65 – 2.66)	0.45	0.88 (0.34 – 2.29)	0.80
No problems opening bottles	566 (91.9)	1			
Medication adherence					
Sometimes/usually misses doses	86 (14.0)	5.99 (3.66 – 9.80)	<0.01	**6.80 (3.97 – 11.64)**	**<0.01**
Never, rarely, very occasionally misses doses	530 (86.0)	1			

Taking high risk medication prior to presentation					
Yes	119 (19.3)	1.73 (1.07 – 2.79)	0.02	1.62 (0.90 – 2.92)	0.11
No	497 (80.7)	1			
Taking an anticoagulant prior to presentation					
Yes	33 (5.4)	1.24 (0.52 – 2.93)	0.62	1.08 (0.30 – 3.88)	0.90
No	583 (94.6)	1			
Taking regular strong opioid prior to presentation					
Yes	37 (6.0)	2.03 (0.97 – 4.24)	0.06	1.56 (0.52 – 4.65)	0.43
No	579 (94.0)	1			
Taking regular insulin prior to presentation					
Yes	23 (3.7)	1.28 (0.46 – 3.51)	0.64	0.76 (0.19 – 2.99)	0.69
No	593 (96.3)	1			
Prescription/s dispensed at more than one pharmacy in previous 6 months					
Yes	158 (25.6)	1.76 (1.13 – 2.73)	0.01	1.49 (0.89 – 2.48)	0.13
No	458 (74.4)	1			
New medication started or medication ceased or dose changed in ED					
Yes	263 (42.7)	3.44 (2.22 – 5.32)	<0.01	**3.91 (2.43 – 6.30)**	**<0.01**
No	353 (57.3)	1			
ED environment/presentation characteristics					
Time of ED discharge					
Out of ‘office hours’	309 (50.2)	0.81 (0.54 – 1.23)	0.33	0.79 (0.49 – 1.27)	0.33
During ‘office hours’ (9am – 5pm)	307 (49.8)	1			
Disposition from ED					
Home	451 (73.2)	1			
Short stay unit	165 (26.8)	1.02 (0.64 – 1.61)	0.95	1.38 (0.80 – 2.38)	0.25
Duration of ED stay					
More than 8 hours	39 (6.3)	2.99 (1.48 – 6.03)	<0.01	**3.23 (1.47 – 7.08)**	**0.03**
More than 4 hrs, up to 8 hrs	140 (22.7)	1.33 (0.82 – 2.17)	0.25	1.37 (0.80 – 2.35)	0.25
Up to 4 hours	437 (70.9)	1			
Change in treating clinician during ED presentation					
Yes	163 (26.5)	1.43 (0.92 – 2.23)	0.12	1.44 (0.83 – 2.49)	0.20
No	453 (73.5)	1			
Time (min) from ED registration to being seen	-	1.002 (1.00 – 1.004)	0.12	1.00 (0.996 – 1.003)	0.83

Note: OR (95% confidence intervals) in bold are significant predictors of MRPs in the multivariable model.

* Pharmaceutical benefit card holders are those receiving income means tested Australian government benefits and entitles patients to more extensive medication cost subsidies than general patients.
