# Supplementary material for: Development of Screening Tools to Predict Medication-Related Problems Across the Continuum of Emergency Department Care: A Prospective, Multicenter Study
Source: Front Pharmacol. 2022 Jul 6;13:865769. doi: 10.3389/fphar.2022.865769 (PMC9299090; doi:10.3389/fphar.2022.865769)
Supplement: Supplementary file 3 [file Table1.pdf]

Appendix 1: Potential predictor variables for which data was collected, handling of missing data and reason for excluding variables from multivariable regression analysis

1. **Patient characteristics**

Variable (missing data noted in brackets)	Description/code	Included in ED Presentation analysis	Included in ED Discharge analysis	Comments: reason for exclusion from multivariable analysis
Age (0)	• Years	✓	✓	Categorized to make incorporation into a screening tool/score simpler.
Sex (0)	• Female: 1<br>• Male: 2	✓	✓	
Pharmaceutical benefit cardholder (pension/concession) (28)	• Yes: 1<br>• No: 0	✓	✓	If missing data: inserted 'No'
Private Health Insurance (0)	• Yes: 1<br>• No: 0	✓	✓	
Interpreter required in ED (0)	• Yes: 1<br>• No: 0	✓	✓	
Communication difficulties: Can the patient describe the medication history in English? (0)	• Yes: 1<br>• No/borderline: 0	✓	✓	Difficulties were due to medical or language reasons
Inpatient in previous 4 weeks (incl SSU admissions) (0)	• Yes: 1<br>• No: 0	✓	-	
Have you seen a specialist in last 6 months? (35)	• Yes: 1<br>• No: 0	✓	✓	If missing data: inserted 'No' (patients with missing data were unsure whether specialist was seen within 6 mo or 6-12 mo, therefore only occasionally seeing specialist)
ED presentation in last month (0)	• Yes: 1<br>• No: 0	✓	-	
Living arrangement (6)	• Home alone, homeless: 1<br>• Home with family/friends/carer, student accommodation, supported accommodation: 2<br>• Residential Care Facility (e.g. health professional manages medication): 3	✓	✓	If missing: inserted 'Home with family' Excluded: due to similarity with 'Who organizes/administers the medicines at home?'

Number of general practice visits in last 6 months (81)	• Number of visits	✓	-	Excluded: most patients had seen GP in past 6 months, but had difficulty quantifying the number of visits
Presenting Complaint: classified as body systems<br>(0)	• CNS: 1<br>• Respiratory/ENT: 2<br>• Cardiovascular: 3<br>• Musculoskeletal/Skin-soft tissue: 4<br>• Gastrointestinal/liver: 5<br>• Genitourinary /gynae: 6<br>• Toxicology: 7<br>• Psychiatry: 8<br>• Oncology/hematology: 9<br>• Ophthalmology: 10<br>• Other (endocrinology, infectious diseases, allergies, dental): 11	✓	-	Excluded: placing complaints in categories did not discriminate between complex cases and simple cases.<br>e.g. gastroenteritis and fulminant liver failure were in the same category. Also, several presenting complaints overlapped between one or more categories – concern about inter-rater reliability.
Discharge Diagnosis: classified as body systems<br>(0)	• CNS: 1<br>• Respiratory / ENT: 2<br>• Cardiovascular: 3<br>• Musculoskeletal: 4<br>• Gastrointestinal / liver: 5<br>• Genitourinary / gynae: 6<br>• Toxicology: 7<br>• Psychiatry: 8<br>• Oncology/hematology: 9<br>• Ophthalmology: 10<br>• Other (endocrinology, infectious diseases, allergies, dental): 11	-	✓	Excluded – as above

2. Medication characteristics

Variable	Description/code	Include in ED Presentation analysis	Include in ED Discharge analysis	Comments: reason for exclusion from multivariable analysis
Potential medication-related reason for ED presentation? (0)	• Yes: 1<br>• No: 0	✓	-	
Number of regular medicines taken prior to presentation (0)	• Used as a marker of the number of medicines taken after discharge	✓	✓	Categorized to make incorporation into a screening tool/score simpler.
Who organizes/administers the medicines at home?<br>(0)	• Self (nil meds): 1<br>• Self: 2<br>• Carer, 'Carer and self': 3<br>• Health professional, 'health professional and self': 4	✓	✓	
Self-reported medication allergies/intolerances (4)	• No allergies or insignificant allergies/intolerances (ie: low risk, patient safe to receive the drug again): 0<br>• Moderate (prescribe with caution) or high (do no prescribe again) risk allergies: 1	✓	-	If missing data: inserted 'No'
Does the patient/carer have problems reading information on medicine labels? (12)	• No: 0<br>• Yes, due to literacy or vision issues: 1	✓	✓	If missing data: inserted 'No'
Patient/carer problems opening bottles (0)	• Yes: 1<br>• No: 0	✓	✓	
Patient reported adherence issue – "People often have difficulty taking their pills for one reason or another. About how often would you say you miss taking your medicines?" (0)	• No (Never/rarely/very occasionally/doesn't take any regular medication): 0<br>• Yes (Sometimes/usually): 1	✓	✓	
Was the patient taking any high-risk medicines prior to admission? (0)	• Yes: 1<br>• No: 0	✓	✓	Defined as: Anticoagulant, insulin, regular strong opioid, glucocorticoid, Parkinson's medication, oral chemotherapy, immunosuppressants

Anticoagulant prior? (0)	• Yes: 1<br>• No: 0	✓	✓	
Insulin prior? (0)	• Yes: 1<br>• No: 0	✓	✓	
Regular strong opioid prior? (0)	• Yes: 1<br>• No: 0	✓	✓	
Number of pharmacies who have dispensed a prescription in the last 6 months (19)	• No pharmacies or 1 pharmacy only: 0<br>• More than one pharmacy: 1	✓	✓	If missing data: inserted “No pharmacies or 1 pharmacy only”
Was a new medicine Rx or dose changed on discharge? (0)	• Yes: 1<br>• No: 0	-	✓	
Dose administration aid (0)	• No: 0<br>• Yes, packed by self/carer: 1<br>• Yes, packed by health professional: 2	✓	✓	
Hearing problems in ED (0)	• Yes: 1<br>• No: 0	✓	✓	Excluded: Difficult to assess over phone because patients often had hearing aids in when at home or on ward (but often not available in ED)
Swallowing problems (0)	• Yes: 1<br>• No: 0	✓	✓	Excluded: Very small number of patients had problems

3. ED presentation characteristics

Variable	Description/code	Include in ED Presentation analysis	Include in ED Discharge analysis	Comments: reason for exclusion from multivariable analysis
Time of ED registration (0)	• Office hours (9am-5pm): 1<br>• Non-office hours (5.01pm – 8.59am): 2	✓	-	
Time of ED Discharge (0)	• Office hours (9am-5pm): 1<br>• Non-office hours (5.01pm – 8.59am): 2	-	✓	
ED length of stay (0)	• Number of minutes	-	✓	Categorized to make incorporation into a screening tool/score simpler.
Australasian triage category (0)	• 1<br>• 2<br>• 3<br>• 4<br>• 5	✓	-	
Mode of presentation (0)	• Ambulance/emergency service: 1<br>• Self: 2	✓	-	
Time from registration to being seen by clinician (minutes) (0)	• Continuous variable (minutes)	-	✓	
Disposition from ED (0)	• Home: 1<br>• SOU: 2<br>• Ward/ICU: 3	-	✓	Determined whether patient was included in discharge model (only included patients who went from ED to SSU or home)
Change in treating clinician during ED admission (5)	• Yes: 1<br>• No: 0	-	✓	If missing data, inserted ‘No’
Hospital/site (0)	• Each hospital site was assigned a number	✓	✓	Excluded: no significant difference between sites in univariate analysis
ED Presentation Month (0)	• Each hospital site was assigned a category	✓	✓	Excluded: no significant difference between months in univariate analysis
Day of presentation (0)	• Categorized by day of the week	✓	✓	Excluded: no significant difference between days in univariate analysis
